# A Scoping Review on the Coping Strategies Used by Intimate Partner Violence Survivors

**DOI:** 10.3390/ijerph22071061

**Published:** 2025-07-02

**Authors:** Xiu Hui Ong, Poh Chua Siah, Qiu Ting Chie, Wan Ying Lee

**Affiliations:** Department of Psychology and Counselling, Universiti Tunku Abdul Rahman, Kampar 31900, Malaysia; siahpc@utar.edu.my (P.C.S.); chie.qiu.ting@gmail.com (Q.T.C.); lwying@utar.edu.my (W.Y.L.)

**Keywords:** intimate partner violence, coping strategies, Skinner’s 11 families of coping, violence against women, scoping review

## Abstract

Intimate partner violence (IPV) is a widespread issue with severe consequences for women’s well-being. This scoping review synthesizes research on coping strategies among female IPV survivors, evaluates measurement approaches, and assesses the applicability of the 11 families of coping framework. Analyzing 27 studies (2017–2022) from the Scopus database, we identified key coping patterns. In response to the first research question, the review revealed methodological diversity, with qualitative interviews predominating (55.56% of studies) alongside quantitative measures such as the Brief-COPE and IPV Strategies Index. All documented coping strategies were successfully categorized using Skinner’s framework, demonstrating its comprehensive utility for IPV research. This complete categorization directly answers our second research question, confirming the framework’s effectiveness for classifying IPV coping strategies. By using this framework, we identified key coping patterns, with seeking social support emerging as the most prevalent strategy (88.89% of studies), followed by escape–avoidance (55.56%) and problem-solving (44.44%). The findings underscore the value of adopting a standardized classification system to enhance consistency across studies and improve comparative analyses. The study contributes to theoretical development by validating Skinner’s model in IPV contexts and offers practical guidance for future research design. By demonstrating the universal applicability of the 11 families of coping, this scoping review provides a foundation for systematic investigations of coping mechanisms and informs targeted support interventions for survivors.

## 1. Introduction

### 1.1. Background of Study

Intimate partner violence (IPV) is one of the most common forms of violence against women, encompassing physical violence, sexual violence, stalking, and psychological aggression by a current or former romantic partner [1,2]. Globally, an estimated 27% of women aged 15–49 have experienced IPV at least once in their lifetime, with prevalence rates varying significantly by region. Sub-Saharan Africa and Southeast Asia report the highest rates (33%), followed by Northern Africa (30%) and the Americas (25%), while high-income regions such as Europe show lower rates (16–23%) [3]. Beyond its immediate physical and psychological harm, IPV perpetuates a cycle of violence, as children exposed to domestic violence are more likely to become either perpetrators or victims later in life [4,5].

To manage the trauma of abuse and its aftermath, survivors employ various coping strategies which can range from problem-solving approaches to emotional regulation [6]. Coping is a dynamic, multidimensional process influenced by individual and situational factors [7]. According to Lazarus and Folkman’s Transactional Model of Stress and Coping, individuals assess stressful situations, select coping strategies, and evaluate their effectiveness before deciding whether to adjust their approach [8]. This model highlights the adaptive nature of coping, where outcomes shape future responses.

### 1.2. Research Gaps

Despite extensive research on coping mechanisms, a significant gap remains in how these strategies are systematically classified for IPV survivors. Existing frameworks often rely on oversimplified distinctions, such as problem-focused versus emotion-focused coping, which fail to capture the complexity of real-world responses. For example, a strategy like “planning” can serve both problem-solving and emotional-regulation functions, making it difficult to categorize under traditional models. Additionally, the lack of a standardized classification system has led to inconsistencies across studies, with overlapping or vaguely defined coping strategies complicating comparative analysis [9].

This study addresses these limitations by applying the 11 families of coping, a theoretically grounded and empirically validated system designed to minimize overlap while accommodating diverse coping behaviors. Unlike previous models, this classification provides clear, mutually exclusive categories, ensuring more precise analysis [10]. The framework excludes only one of the original 12 families—delegation—as it applies exclusively to children, leaving 11 categories relevant to adult IPV survivors.

### 1.3. Skinner et al. (2003)’s 11 Families of Coping

The 11 families of coping are defined as follows [10]:

Accommodation involves adjusting personal expectations to situational constraints through strategies like cognitive restructuring, minimization, and acceptance. Escape encompasses disengagement from stress via cognitive avoidance, denial, and wishful thinking. Helplessness reflects surrender of control through passivity, confusion, and pessimism. Information seeking includes educating oneself about the stressful situation through monitoring and observation. Negotiation represents compromise attempts through priority-setting, persuasion, and deal-making. Opposition involves externalizing behaviors like anger, aggression, and blaming others.

Problem-solving incorporates active approaches including planning, logical analysis, and persistent effort. Self-reliance focuses on internal regulation through emotional and behavioral control. Seeking support entails reaching out to various networks for instrumental help or emotional comfort. Social withdrawal involves isolating oneself through avoidance and concealment. Submission represents relinquishing control through rumination and intrusive thoughts.

**Aims of the study**

Based on the comments that many coping strategies have been identified due to the different measurements used, it is assumed that similar comments can also apply to studies on coping in the subject of IPV [9]. Accordingly, this scoping review aimed to review recent literature on IPV to examine various coping and measurements used by researchers in these studies. In addition, we also aimed to examine whether various coping strategies used by IPV survivors can be classified based on the 11 families of coping [10]. This framework was proposed by Skinner et al. due to the extensive number of coping strategies reported and thus underscored the need to have a framework to classify these various coping strategies. This framework was particularly suitable for our study because, like the broader literature Skinner et al. addressed, IPV research has similarly documented a wide variety of coping strategies requiring systematic organization.

Accordingly, the research questions of this scoping review are as follows:

RQ1: What are the coping strategies used by female IPV survivors?

RQ2: What are the coping measurements used in IPV research?

RQ3: Can the different coping strategies used by women IPV survivors be categorized into the 11 coping strategies as proposed by Skinner et al. [10]?

## 2. Materials and Methods

This scoping review was conducted in accordance with the PRISMA guidelines for reporting systematic reviews and meta-analyses. We have adhered to the PRISMA 2020 checklist and flow diagram to ensure transparency and completeness in our reporting. Specifically, the PRISMA flow diagram illustrates the study selection process, including the initial search results, the number of studies included and excluded at each stage, and the reasons for exclusion. The PRISMA checklist, available at https://www.prisma-statement.org/prisma-2020-checklist (accessed on 18 April 2025), guides us in reporting all essential aspects of the scoping review, including the research question, search methods, inclusion and exclusion criteria, data extraction, and synthesis of findings.

This scoping review on the coping strategies used by female IPV survivors was conducted to gather evidence in the literature in the field of psychology from the Scopus database published between 2017 to 2022. This scoping review was guided by the six-stage methodological framework [11], which includes (i) identifying the research question, (ii) identifying relevant studies, (iii) selecting eligible studies, (iv) charting of data, and (v) collating and summarizing the results.

**Identifying Relevant Studies**

The search for articles was limited to several qualifying criteria. Only journal articles that were written in English and were published on the electronic database Scopus were selected for consideration. The Scopus database was selected as it has a larger dataset with a wider journal range [12] and has a search option that allows users to better identify the material they require based on specific requirements [13]. While other databases such as Web of Science, PsycINFO and PubMed were considered, Scopus was ultimately selected as it had a wider overall coverage and indexed a greater number of sources not indexed in other databases [14,15]. The articles were then further filtered to those published between 2017–2022.

Two keywords were used in the search of articles, which are “coping” and “intimate partner violence”, and only articles published between the years 2017 to 2022 in the field of psychology were selected. This allowed for the search of articles to be general enough that no potentially relevant articles were missed out on. After searching the keywords “coping” and “intimate partner violence”, 297 articles were identified as relevant studies.

**Selection of Eligible Studies**

The 297 articles were reviewed by the researchers to ensure the content of the articles was relevant to the aim and research question of this study, which was to investigate the coping strategies used by women IPV survivors. Further eligibility criteria were applied to ensure that the selected articles were able to provide the necessary information relevant to the study.

The inclusion criteria for this study were as follows: (1) participants must be adult or adolescent women who have experienced intimate partner violence (regardless of marital status)—studies including both male and female participants were eligible, provided they included female IPV survivors; (2) articles must report on the specific coping strategies used by IPV survivors; (3) articles must be original research papers; and (4) articles must be published in English in the Scopus database from 2017 to 2022 (which was when the scoping review was conducted).

The exclusion criteria for this study were as follows: (1) articles outside the psychology discipline; (2) studies where female IPV survivors were not the primary sample; (3) research that did not specifically examine coping strategies among female IPV survivors; (4) articles that are reviews of prior literature; and (5) articles not published in English in Scopus between 2017 and 2022. These exclusions ensured the scoping review focused exclusively on relevant psychological research about women’s coping mechanisms for IPV during the specified timeframe.

**Figure 1:**
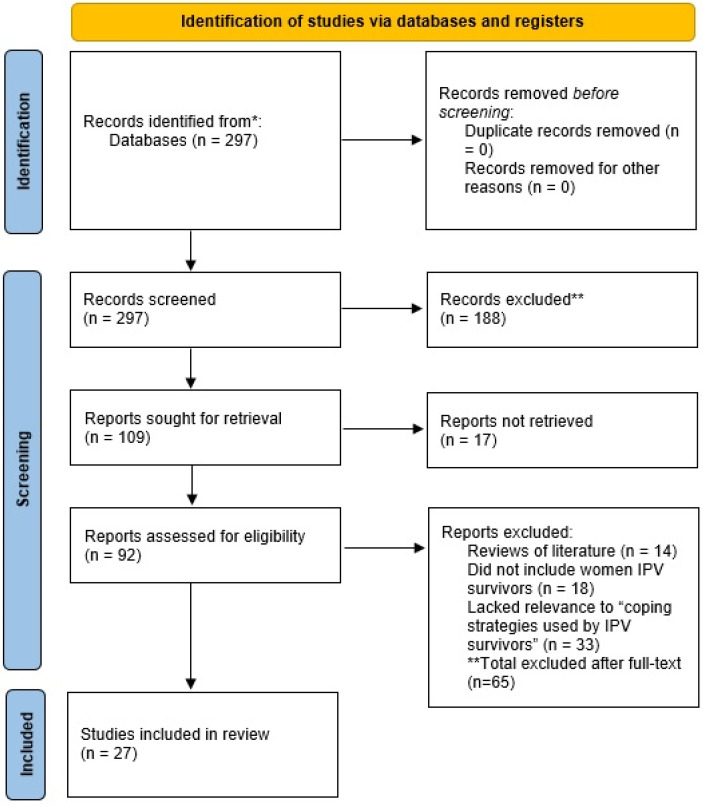
Screening process of articles.

Among the 297 articles identified as relevant articles, two reviewers conducted a full-text assessment of 95 articles. After applying the inclusion and exclusion criteria, 65 articles were excluded for the following reasons: 14 were literature reviews, 18 did not focus on female IPV survivors, and 33 lacked relevant data about coping strategies. Ultimately, 27 studies met all eligibility criteria and were included in the scoping review. The screening process is shown in Figure 1.

**Charting the Data**

A table (Table 1) was constructed to facilitate the charting of data. Table 1 consisted of the necessary information, which included the title of the articles, the design of the study, the method by which the data was collected in the study, sample size and basic demographic information of the sample, the country in which the study was conducted in, and relevant findings that could answer the research question of this study.

**Data Synthesis**

Due to the heterogeneity in measuring coping strategies among IPV survivors, a narrative synthesis was conducted, which included summarizing results in structured tables that compared study designs and findings (see Table 1) and thematically analyzing consistencies and contradictions across studies. Codes regarding coping strategies were generated from the articles reviewed.

**Terminology Clarification**

To ensure conceptual clarity, we adopt the following definitions throughout this study: (1) ‘Coping strategies’ refer to specific behaviors or cognitive efforts survivors employ to manage IPV-related stress (for example, support seeking, escape); (2) ‘Measurements’ denote the tools/methods used to assess these strategies (for example, Brief-COPE, interviews); and (3) ‘Systems’ describe overarching classification frameworks (for example, Skinner’s 11 families). These terms are mutually exclusive and applied consistently across analysis and reporting.

## 3. Results

A total of 27 articles that were published between 2017 and 2022 met the inclusion criteria. The included articles are as below in Table 1.

### 3.1. Coping Strategies Used by Survivors

As shown in Table 1, different coping strategies have been reported, such as social support from family and friends, having safety plans, engaging in self-blame, physically resisting the abuser, and many more.

### 3.2. Measurements Used in IPV Research

As summarized in Table 1, different researchers chose to use different measurements to collect data on survivors’ use of coping strategies. Among the 27 studies, 18 studies used the qualitative research method, 7 studies used the quantitative research method, and 2 studies chose to use a mixed-method. For the studies that used the qualitative research method, 15 of them chose to interview survivors on their experiences and the coping strategies they used (55.56%), while 2 other studies used focus groups to collect data (7.41%). Besides that, one study chose to collect data using virtual ethnography by exploring social interactions occurring in online or digital environments (3.70%) [33].

For the studies that used the quantitative research method, three studies chose to develop their survey items (11.11%), while one study used the Coping Orientation to Problems Experienced Inventory (Brief-COPE) (3.70%) [24]. Other measurements used by researchers included the Cognitive Processing of Trauma Scale (POTS) (3.70%) [22], Intimate Partner Violence Strategies Index (IPVSI) (3.70%) [7], Ways of Coping Scale (3.70%) [30] and the Measure of Affect Regulation Styles (MARS) (3.70%) [35]. Two studies chose to use a mixed-method (7.41%), whereby one study used a combination of interviews and the Measure of Affect Regulation Styles (MARS) [35], while another study used a combination of a self-developed survey and interview to collect data (3.70%) [21].

### 3.3. Codes Categorized Under Skinner’s 11 Families of Coping

After reviewing the 27 articles included in the scoping review, the primary researcher extracted data from the articles regarding coping strategies used by IPV survivors and carried out coding of these data. An example of coding is as follows:

*Original text from article: “I kept saying this can’t be happening, this can’t be real.” Ginny could not believe that her partner was becoming verbally abusive. [denial]*

After coding all the coping strategies that were reported in the 27 articles reviewed, the reviewers attempted to arrange these codes in a more systematic way by reframing the coping strategies according to Skinner’s 11 families of coping. Two independent reviewers examined the codes generated and categorized them based on the 11 families of coping. While there were disagreements over the categorization of a particular coping strategy, a third reviewer was brought in to give their opinion, thereby coming to a decision. Overall, all the codes were able to be categorized according to the 11 families of coping, which can be seen in Table 2.

**Accommodation.** Some coping strategies under this family of coping include distraction and cognitive restructuring [16,31,37].

**Distraction.** Some coping strategies reported in the reviewed articles can be categorized under distraction, including filling time with community activities or getting a job [16], engaging in education or employment after leaving the relationship [39], engaging in activities such as cooking, watching television, listening to music, or performing housework [34], and engaging in various physical activities and hobbies [40].

**Cognitive Restructuring.** One coping strategy that can be categorized as cognitive restructuring is attributing a new identity to the abusive partner [28]. Survivors cope with the disgust they feel towards their abusive partner by attributing a new role or identity to the abuser, which puts distance in the relationship between the survivor and the abuser. For example, one survivor re-identified her abusive partner as “the father of my child” rather than “my husband”. This allowed the survivors to redefine the connection they once had with the abuser. In addition to that, the survivors construct a new identity by being involved in new relationships so that they can identify themselves with new roles, building up their self-worth and self-esteem [28,37]. Similarly, women IPV survivors engaged in remarriage to regain the social status they lost through divorcing the abusive partner [17]. Another strategy included helping other women going through similar abusive situations to find meaning and purpose in their lives [31,37]). Survivors also engaged in positive cognitive restructuring [22].

**Escape.** The coping strategies under this category include protective actions by survivors to avoid further provoking the abuser [6], traveling back and forth from the marital and natal home or leaving the marital home [7,18,34], avoiding abusers at certain times or staying with families and friends [36], false hope that the situation would become better and their abusers would stop abusing them [20,34], denying the abuse they experienced, blocked feelings related to the abuse [20,24], physical avoidance from their abusive partners by locking themselves in rooms to avoid their husbands when they came home drunk [19] or sleeping in separate beds, staying silent to prevent interacting with their husbands, eating disorders and substance abuse [20,37,38].

**Helplessness.** An example of this category of coping strategy present in this scoping review is disengagement from one’s self [24]. Survivors reported experiencing a disrupted self-image during their exposure to abuse and felt disgusted at their changed selves. As such, they tried to cope with this disgust through alienation from the self. They described how they felt like they were acting out of identity when they could not resist the abuse from the abuser and became estranged from themselves during and after the violence. Women IPV survivors also engaged in silence to not be stigmatized by their community, as abuse is considered a disgrace to women in certain cultures [18].

**Information Seeking.** Information seeking involves survivors constantly monitoring their surroundings and establishing safety plans for security [27], taking precautions such as keeping important contact numbers, stashing money and valuables, and maintaining an escape plan [36], and seeking information to understand control and power dynamics in abusive relationships, enabling them to make sense of their past and move forward [37].

**Negotiation.** Negotiation involves survivors involving family members to intervene and negotiate with the abuser, such as seeking joint meetings between their in-laws and birth family to resolve conflicts with abusive husbands [34], or enlisting their birth family to negotiate with abusive partners to stop the abuse [29]. Additionally, survivors attempted direct negotiation with their partners to halt the abuse [7,39], admitted wrongdoing and apologized to their partners to settle issues [18], or employed strategies such as being submissive and complying with their abusers’ demands to maintain peace and avoid [7,19,36,39].

**Opposition.** Opposition took the form of active resistance behaviors among survivors, such as physically hitting back, verbally resisting or standing up to the abusers, rejecting their apologies, using contraception secretly, and also reporting the abusers’ animal abuse behavior to the police [38]. For example, survivors engaged in opposition strategies by fighting back physically against their abusers and refusing to do what the abuser asked [7].

**Problem-solving.** Different coping strategies from reviewed articles have been identified and categorized as problem-solving. These coping strategies include seeking financial independence to escape the abusive relationships [6,7], being hyper-vigilant of their behavior to avoid being abused [39], actually leaving the abusive relationship or getting a divorce [17,18,29,37,38,39] and trying to gain back control of their lives, whether by becoming self-empowered by being employed to gain economic freedom or taking custody of their children to maintain their children or their relationships [17].

**Seeking Support.** In terms of seeking support, different sources of support have been identified from the articles reviewed, such as advice the women received from their friends and family [18,19], the positive feedback and advice they received from their loved ones [16], intervention from their friends and family when the abuse intensified, or when they felt they were unable to reach out for help from the police, as their husbands’ reputation would be affected [29,32].

Besides that, social media is another important source of social support for coping. For example, survivors were able to share their stories of abuse, receive emotional support, and obtain resourceful information and support from members of Facebook communities of IPV survivors [33]. Besides that, these Facebook groups were also a place of resourceful information and support for the women to report the abuse to the authorities. Focus groups consisting of IPV survivors were also helpful to the women in coping with the trauma, as this allowed the women to share similar experiences and feelings that resulted from the abuse [20,37,40]. Parent groups consisting of IPV survivors with children were also helpful to women in increasing their coping capabilities, as involvement in these groups gave them a sense of release when sharing or listening to experiences of abuse, as well as gaining confidence and hope in knowing how to seek support and communicate with their children. This is further facilitated by the professional assistance given in the parent group, which allowed the women to better understand and process their experiences [21].

Moreover, non-governmental organizations (NGOs), medical centers, or even shelters are another source of social support for screening for abuse, care and guidance [6], and counseling services [19,27,37,40]. Besides that, governmental departments or authoritative bodies also provide social support including frightening the abuser, filing complaints or calling the police to stop them from abusing the women, obtaining orders of protection, or contacting lawyers to settle legal issues stemming from the abuse [6,16,23,32].

Furthermore, religion also offers support to women in various ways, such as praying to God to change their partners’ abusive behavior [18,25], praying for inner peace, praying to seek guidance from God on whether to leave their husbands, confiding in their friends at church, or in pastors and religious leaders about their experiences [25], turning to the Qur’an (Islam holy bible) for wisdom and perseverance to cope with the [29,31,32], and intervention, advice and even spiritual coping strategies from religious leaders [29,31,32].

**Self-reliance.** Self-reliance includes coping strategies such as controlled expression of emotion, self-control, and self-reward to focus on the situation and find solutions [35]. Some survivors tried not to cry during abuse to avoid arguments with the abuser [7]. Others used silence for peace of mind and crying as an emotional outlet to relieve negative emotions [18,34].

**Social Withdrawal.** Social withdrawal includes avoiding sharing their experiences of abuse with friends or colleagues due to fears of privacy exposure [25], avoiding seeking help from family and friends to prevent being blamed for the abuse [37], and avoiding approaching religious imams for help because exposing their husbands’ abuse would harm their reputations and was culturally unacceptable [31].

**Submission.** This category includes coping strategies such as engaging in self-blame to cope with the abuse they experienced [19,24,30], and felt they were at fault which resulted in them being abused by their partner [20].

### 3.4. Other Findings

**Most Frequently Reported Families of Coping.** Based on Table 2, the most reported coping strategy was support seeking (88.89%), with escape avoidance (55.56%) and problem-solving (44.4%) coming in as second and third.

**The Countries.** Out of the 27 articles included, 7 studies were conducted in Asia (Malaysia: 1; India: 3; Hong Kong: 1; Indonesia: 2), 7 in Europe (Poland: 1; Greece: 1; United Kingdom: 3; Spain: 1; Bosnia and Herzegovina: 1), 3 in the Middle East (Turkey: 2; Iran: 1), 2 in the African Nations (Ghana: 1; South Africa: 1), 1 in South Pacific (Fiji: 1), 5 in North America (United States: 5) and 1 in South America (Brazil: 1). Two studies were conducted across nations, with one study being conducted in five European nations (United Kingdom, Greece, Italy, Slovenia and Poland), and another being conducted in the United States, Canada, United Kingdom, Spain and Cameroon.

## 4. Discussion

In a review paper of studies related to coping strategies, it was concluded that a wide variety of different coping measurements and coping strategies have been reported, thereby highlighting the need for a standard coping system to categorize all the different coping strategies [9]. This conclusion may also be applied to coping among women IPV survivors, as other studies on coping strategies regarding IPV have revealed the wide variety of coping strategies used and the lack of a consistent system to define and categorize different coping strategies. As such, this study used the scoping review to review recent literature on IPV to examine the various coping strategies reported in the reviewed literature, as well as the various measurements used by researchers in the studies reviewed. This study also aims to investigate whether the various coping strategies reported in the studies reviewed could be categorized according to Skinner’s 11 families of coping.

For the first research question, our findings indicated that various coping strategies have been used by women IPV survivors and that different terminology has been used to label these coping strategies, which can be seen in Table 1 and Table 2. As prior research has noted [9], there has been a lack of clarity and consensus in defining categories of coping strategies. Our scoping review indicates that this comment also applies to the field of IPV. This lack of consensus in defining categories of coping strategies would lead to problems in understanding the coping strategies used by IPV, as it would be difficult to compare all the different studies to produce a complete picture of the coping strategies used by women IPV survivors. Accordingly, a classification system that can categorize all the different coping strategies is clearly needed.

Our second research question is to examine whether studies relating to the field of IPV used a variety of different coping measurements, as prior research commented that the lack of clarity and consensus in defining categories of coping strategies could be related to the use of different measurements [9]. Our scoping review found that different researchers tend to use different measurements and designs in studies of coping strategies among women IPV survivors. This scoping review found that the most common measurement was the use of qualitative interviews to collect data on the coping strategies used by women IPV survivors. However, different terms have been used to describe some similar coping strategies, such as positive cognitive processing versus positive thinking [16,22], distraction versus filling in time [10,16] and emotional regulation versus controlled expression of emotion [18,35]. Besides that, the results of this scoping review also reported five other different measurements that have been used by researchers to measure coping strategies in quantitative studies, such as the Coping Orientation to Problems Experienced Inventory (Brief-COPE), the Cognitive Processing of Trauma Scale (POTS), the Intimate Partner Violence Strategies Index (IPVSI), and others, thus leading to different coping strategies being reported. Accordingly, a system that can categorize these similar coping strategies would be necessary to solve the clarity and consensus issues highlighted [9].

Our third research question examines whether Skinner’s 11 families of coping can address issues regarding clarity and consensus among coping strategies [10]. Two independent researchers matched the coping strategies from the reviewed studies to these 11 families and found that all strategies from the 27 studies fit within Skinner’s framework, demonstrating its applicability in IPV research. This study makes a significant methodological contribution by demonstrating the comprehensive applicability of the 11 families of coping to IPV research. While previous studies have noted the lack of consensus in classifying coping strategies [9], this study’s systematic categorization of all reported strategies across 27 studies proves Skinner’s framework’s unique value in resolving this issue. The following three key advances emerge: (1) the framework successfully integrates both qualitative and quantitative coping measurements under a unified system; (2) it reveals previously obscured patterns (e.g., support-seeking as nearly universal while problem-solving varies culturally); and (3) it enables direct comparisons across diverse cultural contexts where IPV occurs. This evidence establishes Skinner’s taxonomy as an essential tool for future IPV coping research, addressing critical theoretical and measurement challenges in the field.

Therefore, these 11 families of coping can be considered for use by other researchers in future studies of coping while analyzing their data, whereby researchers can choose to categorize the coping strategies based on the 11 families of coping in quantitative studies or develop a coping measurement that includes these 11 families of coping in quantitative studies, which will be useful in understanding and comparing the coping strategies used by women IPV survivors within different contexts. For example, by using these 11 families of coping, as shown in Table 2, we have found that the most commonly used coping strategies were support seeking (88.89%), with escape avoidance (55.56%) and problem-solving (44.4%) coming in at second and third. These findings indicate the importance of providing more resources for helping IPV survivors receive the social support that they need.

## 5. Conclusions

This study has revealed important findings regarding coping strategies among women IPV survivors, namely that there exists a wide variety of coping strategies used by women IPV survivors as well as a wide variety of coping measurements that lack a consensus and clarity in defining coping strategies. This is consistent with Nabbijohn et al. (2021)’s findings and highlights the issues that exist in research regarding coping strategies, especially in IPV studies [9]. This study has also provided support for the practicability of the use of Skinner et al.’s (2003) proposed 11 families of coping, which was able to categorize the wide variety of coping strategies that were reported in the 27 articles reviewed in this study [10].

## 6. Implications

In terms of theoretical implication, the findings of this study are in line with the transactional theory of stress and coping. Our scoping review indicates that women IPV survivors did use strategies to cope with the abusive threats they faced. Importantly, there is no certain strategy that is most appropriate or most effective, as women IPV survivors use a wide range of coping strategies to protect themselves based on their knowledge and experience of what actions will decrease the danger [7], and also the contextual factors of the situation the women were currently in [39].

One example of how IPV survivors decide what coping strategies will decrease the danger they are in is when the women change their coping strategies depending on whether their husbands are drunk [30]. If the husbands are drunk, the women resort to more aggressive coping strategies and change their coping strategies to being more submissive and placating when their husbands are not drunk. Another example is how women engaged in remarriage after leaving their abusive partners, as Turkish society considers divorced women as losing their status, and remarriage allows them to regain this status [17].

Accordingly, a coping theory that considers contextual factors should also be included in the transactional theory of stress and coping, which will be useful to explain why certain families of coping strategies are most likely to be used in certain contexts but not others and why certain families of coping strategies are most effective to reduce threats in certain contexts but not others. As suggested, fit and context are the most important components in determining the effectiveness of coping strategies [41].

As our scoping review suggests that the 11 families of coping can be used to categorize most of the coping strategies used by women IPV survivors, we suggest that these 11 families of coping can be integrated into the transactional theory of stress and coping to examine various context factors and outcomes linked to the adoption of these various coping strategies [8,10]. For example, in a culture where abuse is regarded as a disgrace, would women IPV survivors be less likely to use social support as a coping strategy and more likely to engage in helpless coping strategies?

In terms of practical contribution, this scoping review provided further backing that the 11 families of coping is a practical system for categorizing coping strategies in IPV studies [10]. Future studies may design a measurement to access these 11 families of coping for use in quantitative studies and may also use these 11 families of coping as a framework for use in qualitative studies. This helps to better organize and understand the various coping strategies used by women IPV survivors and facilitates the communication between qualitative and quantitative studies.

## 7. Limitations

Nonetheless, the interpretation of the results of this scoping review should be cautious. This scoping review was limited to the Scopus database and English articles; as such, articles in other languages that may be included in other databases may be overlooked. As such, future studies may consider including studies from various databases to further examine the validity of these findings. Besides that, while comparing the global and regional estimates of violence against women by the World Health Organization, with the countries of the current reviewed studies, more studies are needed in the Western Pacific and thus limit the generalization of current findings.

This scoping review chose to only include publications between the years 2017 and 2022. The decision to focus on articles published between 2017 and 2022 was based on several key considerations. First, this five-year timeframe ensured that the scoping review captured the most recent and relevant research available at the time the scoping review was conducted (which was in 2022), providing an up-to-date synthesis of contemporary knowledge in the field. A five-year span was also chosen to strike a balance between including novel research and allowing sufficient time for studies to undergo peer review and academic discussion, thereby enhancing their reliability. Given the extensive time and effort required for scoping reviews—including literature screening, data extraction, and synthesis—limiting the search to 2022 ensured feasibility while maintaining methodological rigor. While studies published after 2022 were not included, future research could expand this timeframe to incorporate newer findings. Additionally, based on the judgment of saturation, the researchers agreed that the final 27 articles are enough for analysis and to examine the RQ.

## Figures and Tables

**Table 1:** Summary of articles.

Article	Background	Country	Sample	Measurement	Results
1. A Qualitative Analysis of the Coping Strategies of Female Victimization After Separation [16].	This study investigated the coping strategies IPV survivors in Malaysia used after separating from their partner.	Malaysia	15 women aged 18 and above	Semi-structured interview	1. Filling in time: 12 women filled their time to distract them from the stress of their IPV experiences, and engaging in community activities made them feel confident to survive without their husbands. They also felt getting a job would help distract them from facing the stressors at home. 2. Positive thinking: 8 women tried to face their stress and problems with optimism and ignoring their husbands’ disruptions. They also found their children as a source of strength. Many found the strength to continue life after leaving their husbands by considering the needs of their children. 3. Seeking formal services: 2 participants sought the help of formal organizations; one received counseling services from the Islamic Religious Department, while the other went to the Welfare Department to obtain social services that would protect her and her children’s rights. 4. Religion: 6 participants felt that religion provided them with tranquility to them and allowed them to face their problems. 5. Sharing problems through non-formal networks: This referred to informal social support. Most of the participants felt that talking about their experiences to their family or close friends, some who have been victims of IPV, alleviated them of their problems. They also felt confident when they received positive feedback and advice that helped them face challenges, especially after separation.
2. Experiences of Divorced Women Subject to Domestic Violence in Turkey [17].	This study examined the experiences of divorced women who experienced IPV in Turkey.	Turkey	13 women aged 22 to 48	Semi-structured interview	Seeking help Reached out for family support, then sought help from the police, and finally went to shelters as a last resort. Six participants sought help from the police but were sent back to their abusive homes. Some women received informal social support from neighbors and community. Professional psychologists helped participants in coping. Divorce All participants sought divorce in the end. Children Children were a motivation for the women to leave, and after leaving, gaining custody of their children was important to the women, as the women felt lost without their children. Economic freedom (after leaving) Employment gave women empowerment, and economic freedom gave women the power to overcome their violent lives. Remarriage As divorce plays an important part in the status that women lose in society, this status becomes restored by remarriage.
3. Coping Strategies Adopted by Migrant Female Head-load Carriers Who Experienced IPV [18].	This study examined the coping strategies among head-load carriers in Ghana, a profession which employs migrant women due to their low employable skills.	Ghana	20 women aged 14 to 23	Semi-structured interview	Five themes of coping were found from interviewing the participants. Interpersonal coping approach 1. Fighting back: This happened during the early days of the abuse, where the women sought to instill hurt in their partners, which only increased the violence. 2. Apologizing, self-explanation and negotiation: After realizing fighting back made things worse, they tried apologizing or discussing with their partners. While for some, this led to a pause in the abuse that did not last long, for others, although the partner would apologize, the abuse never stopped. Intrapersonal coping/Silence: Silence as a coping mechanism occurred only after most participants used the interpersonal coping strategies and realized they did not work. They sought to reduce the violence and prolong the relationship. 1. Walking out of the house in silence: This was an effective preventive and emotional coping strategy for participants, as they could prevent their partner from physically hurting them. 2. Silence and crying as emotional management: Staying silent did not always prevent the abuse. So some participants used silence as a preventive measure and crying as an emotional outlet which helped them relieve their negative emotions. 3. Silence as culturally motivated: Most participants chose to remain silent about the abuse due to traditional views and stigma about abuse. Their culture viewed abuse as a disgrace because it meant the women did not perform her marital duties well. Socio-personal coping 1. Seeking external advice and support: 9 out of the 20 participants had sought advice or support from a friend, family member or the police. 2. Keeping ties with family and friends: While some participants spoke of how their family and friends were a source of strength, some mentioned how seeking help from outsiders resulted in further complications in the situation. Prayers and hope: After trying many coping strategies, 15 out of 20 participants felt their only hope was God. However, as their religion disproved of divorce, their only option was to stay in the marriage and hoped God would change their partners. Leaving the relationship: Only 5 out of 20 participants left their partners, and they viewed this as a last resort
4. Coping strategies of women survivors of domestic violence residing with an abusive partner after registered complaint with the family counseling center at Alwar, India [19].	This study examined the roles informal and formal institutions of justice played in the coping strategies used by women who experienced IPV.	India	299 married women whose ages ranged from below 20 to above 40.	In-depth interview	Participants initially used coping strategies like “normalizing,” “acceptance,” “denial,” “keeping the peace,” or “blaming themselves.” They tried to be submissive in order to protect themselves from further abuse. They continued using these strategies while trying to find the solution to the abuse, until the situation became too much, which was when they started seeking external help. They isolated themselves from social functions and family members, prayed and wished for miracles. Informal social support was important to participants, especially from their parental family. Having this support allowed women to engage in active coping (strategies were not specified). However in some cases, when the women engaged in active coping, they were not met with support from friends and relatives, as these active coping strategies were not welcome in society. Formal social support referred to support from formal institutions. Researchers note that women must first have support from these formal institutions in order to engage in more cognitive and behavioral coping. Participants who reached out to the MSSK (institution in India protecting DV survivors) had access to authority figures and counselors who provided help and support to the women, and the mental support they received from the MSSK allowed them to engage in more cognitive thinking to appraise their situation. However, some women still chose to remain in the relationship.
5. The Road to Resilience: Strength and Coping Among Pregnant Women Exposed to Intimate Partner Violence [20].	This study examined the experiences of pregnant women who were exposed to IPV, as well as the coping strategies they used.	United States	56 women with a mean age of 27.63	Focus groups	Maladaptive coping Rumination on self-guilt and self-blame. Engaging in avoidance and denial (false hope and substance abuse). Social support Women managed to engage in adaptive coping through social support, whether through personal relationships, the community, or professional support such as counseling. Children as a source of strength Understanding the cycle of IPV empowered the women to leave Ending the relationship Participants and service providers noted that safety was a precondition for any coping strategies.
6. An Evaluation of a Parent Group for Survivors of Intimate Partner Violence [21]	This study examined the experiences of women who participated in a 12-week parent group, who have experienced IPV and had children in the US.	United States	15 women aged 27 to 48	Participants completed survey items that were developed for this study, and focused on their perception of support received in the parent group. They were also interviewed as a follow-up.	Having safety plans Social support through parent group gave them a sense of release when sharing or listening. This experience increases their sense of self, which gave them confidence and hope in knowing how to seek support and managing communication with their children. Professional assistance helped women learn better coping methods. Recovery as an ongoing journey that continues over time.
7. The Mediating Role of Cognitive Processing in the Relationship Between Negative and Positive Effects of Trauma Among Female Victims of Domestic Violence [22].	This study aimed to establish the mediating role of multiple patterns of cognitive processing, reflected by the cognitive strategies used to cope with trauma, in the relationship between negative and positive posttraumatic changes in women following IPV.	Poland	63 women aged 19 to 71 years.	Cognitive Processing of Trauma Scale (CPOTS)	Resolution/acceptance Downward comparison Regret: thinking persistently about what could have been achieved to avoid the events surrounding the trauma Positive cognitive restructuring Researchers note that coping strategies used by individuals may change over time due to the complexity of the cognitive trauma processing.
8. Driving Factors and Actions Taken by Women to Confront Violence: Qualitative Research Based on Art [23].	This study aimed to analyze the critical path of women in coping with situations of violence in Brazil.	Brazil	11 women aged 18 to 59.	In-depth interview	Taking action against abusers: reporting the aggressor, requesting restraining orders, Social support: Seeking help from the Guardianship Council; Seeking help from family members False hope and denial Hoping the abuse would become better Reuniting with the aggressor, hoping things would change Leaving the relationship Researchers noted that participants began the trajectory of confronting the violence when it intensifies in terms of frequency and severity, and that severe physical violence increases the probability of women reporting violence and seeking help in comparison with women who are not severely beaten.
9. Domestic Violence, Social Support, Coping and Depressive Symptomatology among South Asian Women in Hong Kong [24].	This study examined the relationships between IPV, coping strategies, perceived social support, and mental health outcomes among South Asian women in Hong Kong.	Hong Kong	131 South Asian migrant women in Hong Kong aged 18 and above	Brief COPE scale	Maladaptive coping: Self-distraction, behavior disengagement, self-blame, and denial Social support from family and friends
10. Help-seeking behaviors and practices among Fijian women who experience domestic violence: An exploration of the role of religiosity as a coping strategy [25].	This study focuses on help-seeking behaviors and practices among Fijian women	Fiji	18 women aged 18 and above	Interview	Role of religion. Confiding in their pastor and/or religious leader or friends in the church, in seeking help and/or a solution to their problem. Strong personal relationship to God: their faith to God which is maintained by communicating to God through prayers, reading the Bible and personal quiet time with God. This gives meaning and value to their lives in spite of the abuse, adversity, and hardship. Family and friends. When confiding to their family and friends, some of the women noted that they were advised to seek formal help to end the abuse. However some participants revealed that they could not share their experiences with her colleagues and friends, as they might reveal them to other people and everyone would hear about their problem. Formal sources of support. In this study, only a few women reported seeking help from formal sources such as the police and the social service agency. This could be due to personal beliefs about preserving the family unit and the belief that the social service agency may separate the family, and also advice from authorities such as the police who may try to persuade the women from taking action against their abusive spouses. But some women noted that some social agencies would help the women by providing them with information and help on how to escape the abuse.
11. Violence and Abuse in Rural Older Women’s Lives: A Life Course Perspective [26].	This study examined the experiences of older women who experienced IPV in the US.	United States	10 rural older women aged 54 to 70 who had experienced IPV within 5 years of the study	Interview	Denial: One woman described being in denial that her partner was abusive. She eventually left the relationship after realizing there was no changing the relationship Fighting back: Eight women learnt from past mistakes in previous relationships, and refused to be subservient to their abusive husbands. Fought back by being physically aggressive themselves, talking back, and yelling. Fought back by using avoidance behaviors; avoiding conflicts with her husband, locking herself in the room Leaving the relationship: Only four women were completely separated from their partners Social support from the Women’s Resource Center: Nine women used this resource and received necessary help to navigate out of the abusive situation. Women’s shelter was a supportive place in terms of resources.
12. Domestic Violence during the Time of the COVID-19 Pandemic: Experiences and Coping Behavior of Women from Northern Greece [27]	This study investigated the experiences and coping behaviors of abused women from northern Greece during the COVID-19	Greece	15 abused women from Northern Greece aged 30 to 50	Interview	Social support from formal social and counseling services Survivors increased their awareness by learning more about their situation Established safety plans Counselors helped women de-blame themselves, find their lost self, and increase autonomy, competence, self-confidence, and self-esteem and eventually reclaiming control of their lives
13. The Experience of Disgust in Women Exposed to Domestic Violence in Turkey [28]	This study investigates how women experience disgust during and after IPV, as a prolonged and repeated traumatic experience, and how they try to cope	Turkey	6 women aged 18 to 55	Semi-structured interview	Avoidance from perpetrator. The women reported aversion and strong urge for being distant from the perpetrator during the violence process, including when they were faced with coercive attitudes from their husbands. They tried to avoid them by physical separation, such as by separating their beds. Re-identification of the perpetrator with substitutive identity. Even when they became distant from the perpetrator through separation, they still reported disgust related to their connectedness feelings with their perpetrators. They tried to cope with this disgust by reappraising their connection with the perpetrator. They attributed a new role, or identity, to the perpetrator in order to redefine their connectedness. Alienation from self. The women experienced avoidance from a disrupted self-image during exposure to abuse. In these times, they tried to cope with disgust through detachment. Detachment was seen as an alienation feeling from self. They mentioned that they felt like they were acting outside of their identity when they could not resist abuse from the perpetrator. They became estranged to themselves during and after the violence. Re-identification of self with new relationships. After ending an abusive relationship, they still experienced self-disgust as a result of a disrupted self-image during violence. To repair this image, they started new relationships that they could identify themselves in with new roles. Thus, they could disconnect with the self in trauma and repair the distorted self-image.
14. Experience and coping strategies of women victims of domestic violence and their professional caregivers: a qualitative study [6].	This study examines the strategies which had been used by women who had been victims of IPV in Tehran, Iran	Iran	12 women survivors (mean age of 35.6) and 14 women medical professionals (mean age 39.7) who had worked with survivors	Semi-structured interview	Pragmatic actions Asking for help, financial independence, protective behaviors, spouse conviction, legal prosecution process, and securing the environment Social support: Some participants sought help from professionals and organizations, including medical services and counseling. Support from family/friends Denial of the abuse/trying to remain hopeful: Some women stayed in the relationship because they remained hopeful things would change or had misplaced trust in the abuser. Some women chose not to react to the abuse, however the researcher did not further elaborate on this.
15. Resistance Strategies of Madurese Moslem Women Against Domestic Violence in Rural Society [29].	This study examined the resistance strategies used by Madurese women in Indonesia.	Indonesia	4 Madurese women who were victims of IPV	Semi-structured interview	Internal resistance strategies This strategy referred to prayers and religion, which they hoped would give them inner peace to cope with the abuse. External resistance strategies These are when the women resorted to outside help to deal with the situation. Some participants received support and help from friends and families, as well as the police, which in some cases stopped the abuse. Some sought the help of shamans and religion, and while some filed for divorce, they eventually withdrew the request out of shame and stigma. Another participant sought the intervention from formal institutions in the form of divorce. It must be noted that the resistance strategies mentioned in this study were more ways to stop the abuse, rather than coping strategies.
16. Through the Life of their Spouses- Coping Strategies of Wives of Male Alcoholics [30].	This study examines the coping strategies used by wives of alcoholics in India in order to cope with the violence they face from their husbands due to their drinking habits.	India	Indian women above the age of 20, whose husbands were alcoholics. The number of participants was not specified.	Ways of Coping Scale	Avoidance Competition Taking special actions Seeking support Adjusting their expectations Denial Self-blame * No further elaboration was given by the researchers regarding the coping strategies reported by the women. The wives of alcoholics adopt the coping strategies to avoid divorces in the marriage, as the divorces will hamper the future of their children too
17. Black Muslim Women’s Use of Spirituality and Religion as Domestic Violence Coping Strategies [31].	This study examined the use of religion and spirituality of the black American Muslim community in coping with IPV.	United States	6 black Muslim women	All participants completed an initial interview, with another 5 completing a follow-up interview	Seeking help from religious leaders. The women sought out the help of the local imam (religious leader) and some received advice, refuge and help from them. Some visited the homes and intervened, asking the husbands not to abuse the women. However, despite the help the religious leaders gave the women, the imams still advised the women to stay in the relationship. Some women were afraid to approach the religious leaders, as it is the culture and belief that exposing their husbands’ actions would affect their images, and the community was not accepting of this. Spiritual coping strategies: Prayer. Most women turned to prayer for guidance and support, and to fix the problems in their marriage. Some also turned to prayer in order to seek guidance as to whether they should leave the relationship. Using Qur’anic wisdom. The women sought strength to persevere and strategies to escape the abuse from the Qur’an. Some believed that the Qur’an provided them wisdom and saved their lives from their husbands’ violence; one woman escaped being strangled to death by her husband by invoking her God’s name, which stopped her husband from further abusing her in that moment. Spiritual cleansing. This was specific to one participant’s experiences, whereby her husband sought to harm her by use of voodoo. She would then take special baths to protect herself and her children from the voodoo curses her husband placed on her. Connecting to a larger purpose. Participants connected their experience of abuse to having a larger meaning and purpose in their lives, and they tried to help other women going through the same experiences, and this gave them the power that the abuse has taken from them.
18. Black Muslim Women’s Domestic Violence Help-Seeking Strategies: Types, Motivations, and Outcomes [32].	This study examined how black Muslim women in the US sought help for IPV.	United States	6 black Muslim women	Interview	Family and friends. One participant sought help from family and friends when her husband left a physical mark on her from the abuse, as the abuse intensified. Other sought out family and friends to deal with the abuse because they wanted to preserve the image of their husband, which would be affected if they reported the abuse to the police. Religion and spirituality. Services. Four participants sought the help from IPV help hotlines, staying in shelters and receiving counseling. The women usually resorted to this option only when they exhausted all other strategies, and when they realized their survival depended on seeking help from social services. Legal system. Women called (or threatened to call) the police, obtained orders of protection, and contacted lawyers to settle legal issues that stemmed from the abuse, such as monetary disputes and immigration status concerns. They resorted to this option out of fear for their safety, or when other people such as friends asked them to.
19. Beaten Into Submissiveness? An Investigation Into the Protective Strategies Used by Survivors of Domestic Abuse [7].	The aim of the study was to identify the prevalence and perceived helpfulness of a variety of protective strategies that were used by female survivors of domestic abuse in the UK and to explore factors that may have influenced strategy usage.	London, United Kingdom	40 women in outer London aged 18 and above	Intimate Partner Violence Strategies Index (IPVSI)	Placating Tried to keep things quiet for abusers Did whatever the abuser wanted to stop the abuse Tried not to cry during the abuse Tried to avoid the abuser Tried to avoid an argument Resistance Fought back physically Chose to sleep separately from them Refused to do what they said Ended or tried to end the relationship Fought back with words rather than physically Left home to get away from them Informal support Making sure there were other people with them (other than the abuser) Sent children to stay with a relative Talked with family/friends about what they could do to protect themselves and children Stayed with family or friends Legal Filed an application for a protection order Called the police Filed or helped file criminal charges Tried to receive help from legal aid Safety planning Keeping car/house keys close by Keeping money/valuables close by Keeping important phone numbers for help Keeping extra supply of basic necessities for themselves/children Keeping important papers hidden Formal support Talked with someone at a domestic abuse program, refuge, or crisis line Stay at shelter Sought help from religious figure
20. Facebook group types and posts: Indonesian women free themselves from domestic violence [33].	This study examined how emerging virtual communities on Facebook provided support for Indonesian women who experienced IPV.	Indonesia	Members in 3 Facebook communities for women who had experienced IPV	Virtual ethnography: Members’ posts IPV were examined Interviews: Researchers interviewed group moderators and members	Posting about their stories in groups on Facebook provided a safe space for Indonesian women where they received emotional support. They also received information and support to report the abuse to the authorities. They also receive support when other survivors share similar experiences with them.
21. Coping strategies in the face of domestic violence in India [34].	This study examined the coping strategies of abused women in India.	India	21 low-income women aged 18 and above	Interview	Back and forth between marital and natal home. This was a unique strategy for women in India and happened when the abuse started to affect the children and was beyond the tolerance level of the women. Joint meeting. Joint meeting between the woman’s natal and in-laws side of the family was the most common strategy used to deal with the issue of abuse. Sometimes, the negotiations were very specific and worked towards achieving a resolution to day-to-day conflict between the woman and the in-laws’ side of the family. However this was only a temporary solution and the men would go back to abusing the women after. Religion/spirituality. Belief in God, praying and chanting God’s name, or being involved in rituals were different ways in which women kept themselves busy, helping to cope with abuse on a daily basis. Hope, staying quiet, keeping busy. Hoping that the abusive husband will change, staying quiet or crying were other emotion-focused strategies that women utilized to deal with abuse in their lives. Distraction Cooking, watching entertainment sitcoms on the television, performing household work, among others were some of the strategies reported by women.
22. “Mouth Wide Shut”: Strategies of Female Sex Workers for Coping With Intimate Partner Violence [35]	The aim of this study was to explore the relationship between possible violence suffered by female sex workers in their intimate relationships, with their affects, coping strategies, and emotional regulation to overcome such violence and improve their well-being.	Spain	137 Spanish female sex workers who experience IPV	Interview Measure of Affect Regulation Styles (MARS)	Venting Self-reward Self-control Confrontation Direct or indirect verbal confrontation is not functional because it could be associated with greater intensity, displeasure, and less control. Controlled expression of emotion
23. The use of help seeking and coping strategies among Bosnian women in domestic violence shelters [36]	This study examined the coping strategies used by Bosnian women who experienced IPV currently in domestic violence shelters.	Bosnia and Herzegovina	107 women with a mean age of 39 years old	Researchers devised 19 yes/no questions about the women’s use of different strategies in response to the violence they experienced.	Communication strategies Talked to partners about the violence. Talked with family members to end the violence. Creating a code to let others know if in danger. Tried to end relationship. Avoidance strategies Leaving their homes Avoiding the abuser at certain times Avoided seeing or talking with friends or family. However, the participants were split on whether this made the situation better or worse. Protection and resistance Tried not to resist to keep the violence from escalating. Did whatever the partner want to stop the abuse. Most times this made the situation worse. Safety-planning Keeping a list of important numbers. Keeping money and valuables safe. Having an escape plan.
24. Overcoming Abuse: A Phenomenological Investigation of the Journey to Recovery From Past Intimate Partner Violence [37].	This study investigated the lived experiences of survivors of who had overcome abusive relationships and created violence-free and meaningful lives.	US, Australia, Canada, England, Spain and Cameroon	123 participants (117 women, 3 men and 3 unspecified) who experienced IPV above the age of 21.	Researchers developed a survey for this study with questions that asked about participants’ experience of the abuse.	Build support networks Counseling and support groups Recreating and regaining one’s identity post-abuse Using experiences to help others Embracing freedom by embarking on a new career or making their own decisions. Learning about dynamics of abusive relationships and using that knowledge to examine past experiences of abuse. Acceptance of past experiences, forgiveness of self and abuser. Some participants initially resorted to destructive coping strategies, such as consuming drugs and alcohol, while others coped through therapy, friendships, and family support. Participants describe their process of healing as ongoing, with some participants describing it as a grieving process that will take time.
25. ‘I Know it was Every Week, but I Can’t be Sure if it was Every Day: Domestic Violence and Women with Learning Disabilities [38]	This study examined the experiences of women with learning disabilities in the UK who were victims of IPV.	United Kingdom	15 women over the age of 18, with learning disabilities who have experienced IPV	Interview	Verbally resisting/standing up to perpetrators Hitting back Rejecting apologies Using contraception secretly Reporting animal abuse to the authorities Help-seeking from authorities/social services Leaving the relationship: Many women in this study made multiple attempts to leave and of course, the ultimate resistance is to permanently leave the violent relationship, which all participants in this sample did eventually.
26. ‘You just deal with it. You have to when you’ve got a child’: A narrative analysis of mothers’ accounts of how they coped, both during an abusive relationship and after leaving [39]	This study examines the experiences of IPV survivors who were mothers in the UK.	United Kingdom	Eight women from two IPV shelters in the UK, aged 25 to 55 years	Interview	During the relationship Social support: Seeking support from family and friends or seeking voluntary and statutory services. However, the women avoided telling family and friends while the abuse was ongoing, and only told them and received support after leaving. Most women could not describe how they coped, only that they had no choice but “just had to”. Some of the strategies they used helped them forget and deny the relationship difficulties and associated emotions. Active strategies. The women felt they had no option but to actively change their behavior to cope with the challenges they faced. This included calling the police when their ex-partner’s abuse became intolerable or leaving their ex-partner at times before they ended the relationship Pleasing the partner or being hyper-vigilant of their own behavior to avoid being abused. Running away from difficult situations or hiding from their ex-partner A few women reported using alcohol, illegal drugs or over-eating to distract themselves from painful feelings, both during and after the relationship. After leaving the relationship Keeping busy through education or employment After they left the relationship, some women tried to reconcile with what happened and that they had done their best to cope.
27. Women’s experiences of domestic violence and mental health: Findings from a European empowerment project [40]	This article reports on an action-research project adopting a strengths-based approach to recovery funded by the European Commission.	Five European nations: United Kingdom, Greece, Italy, Slovenia and Poland	136 women aged 25 to 62	Data was collected from the training programs through focus groups	The strategies highlighted in this study were aimed at wellness and recovery of women who have left the abusive relationships, and may be considered coping strategies for women after ending the abusive relationships. Women felt that first and foremost, contact with children, family, friends and welfare services contributed to their wellness. Additional responses were going out socially, traveling, focusing on job, helping others, television, reading, painting, sleeping, music, relaxing, eating, shopping, alcohol, smoking, and antidepressants. Physical activities such as dancing, horse riding, cycling, walking and being with nature were cited too. Cleaning the house was mentioned—indicative maybe of women wresting back some control over their environment. A minority expressed a preference for being alone and silence

**Table 2:** Coping strategies according to Skinner’s 11 families of coping.

Skinner’s Framework	Percentage (%)	Coping Strategies
Support seeking	88.89	Support from family and friends (including advice and intervention) [7,16,18,19,25,29,32,36] Seeking support (researcher did not elaborate from whom support was sought) [30] Sought support from neighbors and community [17] Support from other survivors: o Facebook support groups [33] o Focus groups [20,37,40] o Parent groups for survivors with [21] Support from professional and formal organizations that provide medical screening, and care and guidance from medical professionals [6] Seeking help from social services/counseling centers [6,7,19,25,26,29,38] Sought help from government departments/authoritative bodies when it came to their children and their rights [16,23] Seeking help from legal system and services, receiving orders of protection, or contacting lawyers to settle legal issues stemming from the abuse [6,7,23,32] Seeking help from police [7,17,25,38] Religion o Belief in God, praying and chanting God’s name, or being involved in rituals [34] o Provided the women with tranquility and allowed them to face their problems [16] o Faith in God, pray that God will change partners’ behaviors [18,25] o Support from religious leaders or friends from religious settings [25] o Interventions or coping strategies from religious leaders [7,29,31,32] o Turn to prayers for peace and wisdom [29,31,32] o Religion as a deterrent (husband was discouraged from abusing women when invoking the name of God) [31]
2. Escape avoidance	55.56	Tried to avoid abusers, and tried to avoid getting into arguments with abusers [7] Protective actions by survivors to avoid provoking abuser, e.g., leaving home, shouting, protecting head and neck [6] Traveling back and forth from the marital and natal home or the marital home to avoid being at home with the abuser [7,18,34] Avoiding abusers at certain times, or staying with families and friends [36] False hope that the situation would become better and abusers would stop abusing them [20,23,34] Denial (of situation, feelings) [6,20,26,30] Maladaptive self-distraction and denial [24] Physical avoidance from abuser, e.g., avoiding their husbands, locking themselves in rooms when their husbands came home drunk, physical separation by sleeping in different beds [7,19,28] Avoid conflict with abuser and locking oneself in the room to avoid the abuser [26] Maladaptive behaviors such as substance abuse and eating disorders as a form of avoidance [20,37,39,40]
3. Problem solving	44.4	Seeking financial help to leave the abusive relationship [6,7] Being hyper-vigilant of their own behavior to avoid from being abused [39] Gaining control of their lives, through economic freedom, gaining custody of children or relationships [17,27] Leaving the relationship/getting a divorce [6,7,17,18,20,23,29,37,38,39]
4. Negotiation	29.63	Intervention from family to negotiate to stop the abuse [29,34] Personally talking with abusive partners to stop the abuse) [36,39] Admitting they were wrong and apologize just to stop the abuse [18] Being submissive to keep their partners happy, to keep the peace [7,19,36,39] Trying to keep things quiet for the abuser [7] Adjusting expectations [30]
5. Accommodation	29.63	Finding meaning and peace by taking experiences and helping other survivors [31,37] Facing stress with optimism [16] Resolution/acceptance, positive cognitive restructuring, downward comparison [22] Reappraising relationship with abuser to cope with the disgust and negative feelings [28] Rebuild identity by building up self-worth and self-esteem [37] Engaging in activities such as cooking, watching the television, listening to music or performing housework [34,40] Engaging in community activities or getting a job as a distraction [16] Keeping busy through education or employment [39]
6. Information seeking	22.22	Being aware of their situation and having safety plans in case they needed to escape [27] Having precautions to ensure safety, and having an escape plan [21,36] Did safety planning by keeping car/house keys close by, keeping money/valuables close by, keeping important phone numbers for help, keeping extra supply of basic necessities for themselves/children and keeping important papers hidden [7] Educating themselves on their abuse, leading to acceptance and acknowledgement [37] Understanding the cycle of IPV empowered the women to leave [20]
7. Submission	18.52	Self-blame [19,20,24,30] Regret [22]
8. Opposition	14.81	Active resistance behaviors among survivors such as physically hitting back, verbally resisting or standing up to the abusers, rejecting their apologies, using contraception secretly and also reporting the abusers’ animal abuse behavior to the police [38] The women fought back by being physically aggressive themselves, talking back, and yelling [26]. Fighting back both verbally and physically against their abusers and refusing to do what the abuser asked [7] Confrontation [35]
9. Helplessness	11.11	Alienation from self. Women coped with disgust at themselves by detachment from the self. They mentioned that they felt like they were acting out of identity when they could not resist abuse from the perpetrator. They became estranged to themselves during and after the violence [28] Silence to prolong the relationship, not as a form of emotional regulation [18] Survivors engaged in behavior disengagement [24]
10. Social withdrawal	11.11	Avoid telling family and friends about the abuse [25,37] Avoid going to religious leaders for help and intervention [31]
11. Self-reliance	11.11	Controlled expression of emotion, self-control and self-reward [35] Tried not to cry [7] Silence and crying as emotional regulation [18,34]

## Data Availability

No new data were created or analyzed in this study. Data sharing is not applicable to this article.

## Abbreviations

The following abbreviations are used in this manuscript:

IPV	Intimate Partner Violence
POTS	Cognitive Processing of Trauma Scale
IPVSI	Intimate Partner Violence Strategies Index
MARS	Measure of Affect Regulation Styles
WHO	World Health Organization
NGO	Non-governmental Organizations
